# Understanding the histological and histochemical mechanisms involved in gum arabic formation in *Senegalia senegal*, a key species of the Sahelian region

**DOI:** 10.1007/s00709-026-02220-y

**Published:** 2026-06-11

**Authors:** Ali Abdoulkadri Abdoulaye, Frédéric Gatineau, Aichatou Assoumane, Matthieu Dejean, Christelle Baptiste, Jean-Luc Verdeil

**Affiliations:** 1 Faculté des Sciences et Techniques, Université Abdou Moumouni de Niamey, Niamey, Niger; 2 CIRAD, UMR AGAP Institut, PHIV Montpellier, Montpellier, France; 3 UMR AGAP Institut-PHIV, Université de Montpellier, CIRAD, INRAE, Institut Agro, Montpellier, France

**Keywords:** Gum arabic, *Senegalia senegal*, Bark anatomy, AGPs, Companion cells, Schizogenous gum pockets, Secondary phloem

## Abstract

*Senegalia senegal* produces a hydrocolloid known as gum arabic, which is rich in arabinogalactan proteins (AGPs) and of high economic importance. Despite its economic value, the anatomical origin and formation mechanisms of gum arabic remain poorly understood. In this study, we combined histological, histochemical, and immunolocalization techniques to investigate the spatial, cellular, and temporal dynamics of gum formation in *S. senegal*. We show that gum pockets are formed exclusively in the inner secondary phloem, between phloem rays, and are initiated by companion cells. Using Yariv reagent and JIM4 antibody, we identified AGP accumulation as an early hallmark of pocket initiation. Gum pocket development occurs via a schizogenous process, involving progressive cell wall separation at the middle lamella, without signs of cell lysis. We also demonstrate that gum pocket formation is seasonally regulated, peaking during the dry season and followed by a gradual resorption phase. The decline in AGP signal and the formation of new phloem tissues suggest a possible metabolic recycling of non-exuded gum to fuel cambial reactivation. Importantly, the surface area of gum pockets correlates with the amount of gum harvested, offering a potential anatomical proxy for selecting high-yielding trees. Our findings redefine the developmental and physiological framework of gum arabic production and provide new perspectives for genetic improvement and sustainable resource management in Sahelian ecosystems.

## Introduction

Gum arabic is a natural water soluble exudate primarily obtained from the stems and twigs of *Senegalia senegal* (syn. *Acacia senegal*), a leguminous tree native to the arid and semi-arid regions of sub-Saharan Africa. Among the ~ 30 gum-producing tree species identified across dry African zones (Dione 1996), two major groups are recognized: hard gum producers, mainly *S. senegal* and, to a lesser extent, *A. polyacantha*, and crumbly gum producers, such as *A. seyal* (Mallet et al. 2003). In the Sahel region, *S. senegal* and *A. seyal* dominate gum production, with *S. senegal* alone accounting for nearly 90% of the gum arabic traded on global markets (SOFRECO 2022). This complex hydrocolloid, rich in arabinogalactan-proteins (AGPs), is synthesized seasonally in bark tissues under environmental cues and is highly valued for its emulsifying, stabilizing, and gelling properties. Locally, gum arabic also plays roles in food, traditional medicine, and artisanal practices. The gum is produced as a result of a slight incision (tapping) made by harvesters or through cuts made by insects or animal teeth on the bark in the dry season. Two to three weeks after tapping, the tree exudes a sap that gradually hardens and becomes a gum nodule. The gum is then harvested by collectors, who may or may not be equipped with specialized harvesting tools (FAO, 1996; Asgar 2024).

Despite its global economic relevance, gum arabic production in the Sahel remains unstable and poorly managed. The sector faces inconsistent yields, unorganized value chains, and low adoption of best production practices. Gummosis is a natural phenomenon involving the flow of viscous, pale yellow, or golden sap from the bark of the trunk or branches. It is considered to be a defense mechanism used by trees against injury or pathogens (Desfemmes 2026). These issues largely stem from major knowledge gaps concerning the biological and anatomical processes underlying gum biosynthesis in *S. senegal*. Most existing anatomical studies are outdated and do not reflect recent advances in plant developmental biology. Although the molecular and biochemical composition of gum arabic has been characterized in recent decades (Atgié 2018; Thevenet 2009; Nussinovitch 2009), especially the predominance of AGPs, the identity of the specific cells responsible for their synthesis and secretion remains unknown.

Early anatomical observations, beginning with Labat (1728), who hypothesized intracellular gum formation, were followed by more detailed analyses from Prillieux (1875), Lutz (1895), and Ghosh and Purkayastha (1962), who suggested that gum accumulates in inner bark tissues, likely within the phloem. Later, Mouret (1987) and others proposed that gum production may result from cellular degeneration triggered by environmental stress or injury. However, these interpretations were based on outdated studies that relied on traditional staining techniques common at the time, which lacked molecular specificity, and therefore could not confirm the nature or origin of gum components.

To address these limitations, our study presents a comprehensive, modern investigation into the cellular, anatomical, and chemical foundations of gum formation in S. senegal. Specifically, we aim to address the following unresolved questions:

- What is the precise location and developmental timeline of gum pocket formation?
- Which cell types initiate AGP biosynthesis and contribute structurally to gum accumulation?
- Does gum formation result from lysigenic tissue breakdown, or is it governed by a schizogenous, developmentally controlled process?
- What is the fate of gum pockets at the end of the gummosis period, and what happens to the residual, non-exuded gum?

To answer these questions, we employed a combination of histological, histochemical, and immunolocalization techniques. Using Yariv reagent, which is specific to arabinogalactan-protein (AGP) epitopes in general, and the JIM4 monoclonal antibody, which specifically targets epitopes of AGPs in gum arabic, we mapped the spatial and temporal dynamics of gum formation and identified the cell types initiating this process. Our findings reveal that gum pockets form exclusively within the inner secondary phloem, between phloem rays, via a schizogenous mechanism driven by companion cells, without any signs of cellular lysis. We also show that gum pocket formation follows a seasonal cycle, and that residual gum may be resorbed and internally recycled during cambial reactivation.

Finally, our results demonstrate a positive correlation between the surface area of gum pockets and gum yield, as well as between the ratio of gum pocket surface area to inner secondary phloem surface area and the quantity of gum harvested. These findings highlight the potential of anatomical markers for selecting high-yielding individuals. Overall, this study provides novel insights into the cellular mechanisms of gummosis in S. senegal and lays the foundation for future breeding programs and sustainable gum arabic resource management in Sahelian ecosystems.

## Material and method

### Plant material and sampling procedure

The biological material studied consisted of pieces of *Senegalia senegal* cylindrical shoots fragments (length: 5 to 6 cm, diameter: 4 to 5 cm) collected from 3 healthy adult trees exhibiting gum nodules or signs of exudation, spaced at least 30 m apart.

For each tree, three branches estimated to be 2–3 years old (the typical age for gum production) were selected, each located in a distinct radial direction and forming an angle of ~ 140° relative to the trunk axis. Sampling height was standardized between 1.5 m and 3 m above ground level.

Sampling was conducted during three distinct periods at the Korahane site (14°35’43”N, 06°33’29”E) in the Sahelian zone of Niger, characterized by annual rainfall of 300–350 mm and temperatures ranging from 14 °C (January) to 45 °C (May): Non-gummosis period (October), Gummosis period (March): (samples were taken from areas of the same branches showing active gum nodules), end of gummosis (April): (samples were collected from branch areas showing residual gum traces).

Three samples were collected at different locations spaced 15 cm apart, resulting in 9 samples per tree (3 branches × 3 samples). This design yielded 27 samples (3 trees per period) and 81 samples across the three periods.

### Sample processing

For conventional histology, bark samples collected in the field were immediately fixed as previously described (Buffard-Morel et al. 1992) for a minimum of 2 days at 4 °C, by immersion in a 0.2 M phosphate buffer solution containing 2% (w/v) paraformaldehyde, 1% (w/v) caffeine, and 2% (v/v), glutaraldehyde 25% until transported to the laboratory. Samples were then subjected to vacuum infiltration for 2 h, alternating between 15 min of vacuum and 15 min at atmospheric pressure. After vacuum treatment, samples were rinsed in phosphate-buffered saline (PBS buffer) for 15 min and then sequentially transferred to 50% ethanol for 15 min and then transferred to 70% ethanol for storage at 4 °C.

For immunolocalization, samples were fixed in 1X PBS buffer containing 4% (w/v) paraformaldehyde for a maximum of 16 h at 4 °C in dark, (Roongsattham et *al.*, 2016). After fixation, samples were washed twice in 1X PBS buffer containing glycine (0.1 M), followed by two washes in 1X PBS buffer. Samples were then dehydrated in ethanol 50% for 1 and 2 h followed by 70% EtOH for 1 and 2 h at room temperature. Samples for histology or immunolocalization were preserved in 70% ethanol at 4◦C until further processing.

Bark samples were sectioned using a vibratome (Microm HM 650 V, Walldorf Germany). Cross Sect. 20 μm thick were obtained from on each sample, extending from the wood to the suber. The sections, ready for histological staining and immunohistochemical labeling, were preserved in 70% ethanol at 4 °C.

### Histological staining procedures

For the anatomical and histological characterization of the bark, sections were stained with toluidine blue (pH 4.6). For histochemical analysis, several staining techniques were employed to identify tannin-containing cells and to localize specific compounds within the bark tissues: Stiasny reagent combined with FeCl₃ (Haida et al. 2021) and vanillin-HCl were used to detect catechin-type and gallic tannins (Schofield et al. 2001; Yajun et al. 2009), Oil Red was applied to reveal hydrophobic lipids in the phellogen zone (Acosta 2018), phloroglucinol was used to highlight lignified fibers (Pomar and al. 2002), Yariv reagent was employed to detect arabinogalactan proteins (β−1,3-galactan motif) (Kitazawa et al. 2013; Saladino et al. 2020), and Lugol’s solution was used to identify starch reserves (Hinchman 1973).

### Immunolocalization

Cross-sections were first incubated overnight in glass cups containing a blocking buffer solution (5% bovine serum albumin (BSA) in 1X PBS buffer (pH 7.4)) with gentle shaking at room temperature.

Sections were then removed from the blocking solution and incubated for 24 h with the primary antibody (JIM4 or LM26 Table 1) under the same temperature and agitation conditions. After incubation, the sections were washed three times for 15 min each in 1X PBS with gentle agitation.

**Table 1:** Primary Antibodies used for immunolocalization of epitopes of interest

Antibodies	Target/epitope	Source/Host	Dilution*	Provider	References
LM26	β-(1,4)-galactans	Rat	2/100	Laboratory of Paul Knox, University of Leeds	Torode et al. (2018).
JIM4	β -GlcA-(1,3) - α-GalA-(1,2)-Rha	Rat	2/100	CarboSource	Yates et al. (1996)

*LM* Leeds Monoclonal, *JIM* John Innes Monoclonal, * dilution from commercial solution

Following this, sections were incubated with the secondary antibody (ACII) (diluted to 0.2% in 1% PBS buffer). Alexa Fluor 546 Goat Anti-Rat was used as ACII for detection in both cases. After three rinses with PBS (pH = 7.4) the samples were mounted between slides and coverslips using a glycerol water mounting medium for observation.

### Microscope observations

Histochemical staining was observed under a wide-field epifluorescence microscope (NIKON Eclipse Ni-E Tokyo, Japan) equipped with a Nikon CMOS DS-Ri2 color camera with a PLAN APO 2 × 0.1 NA objective lens. Brightfield imaging was performed with transmitted white light illumination, and images acquired using NIS-Elements software. The main objectives used were as follows: 4X (Apo plane λ 0.2 NA), 10X (Apo plane λ 0.45 NA), 20X (Apo plane λ 0.75 NA), 40X (Apo plane λ 0.95 NA), and 100X (Apo plane λ 1.45 NA oil). The filter cubes used included DAPI longpass UV-2 A (330–380 nm excitation, 400–420 emission and GFP longpass B-2 A (450–490 excitation, 500–510 emission).

Immunohistochemical preparations were observed using a Zeiss LSM-880 confocal multi-photon microscope equipped with various types of lasers (405 nm, Argon (458, 488, 514 nm), and a Chameleon (Coherent Ultra II tunable laser 680–1080 nm). The objectives used were 10X (plane Apo 0.45 NA), and 40X (LD C-Apo 1.1 NA W)). The system allows acquisition of XYZ, XZY, lambda, and mosaic images. Images were captured using ZEISS ZEN Black software.

### Images processing and analysis

Custom macros were developed in ImageJ to assist quantitative analysis of bark tissues while retaining expert validation. One macro guide manual measurements and contouring: users select a category (e.g., “Fiber”), perform the defined number of measurements, and add them to the ROI Manager results are exported in Excel format. A second macro enables semi-automatic detection of gum under specific staining conditions (Toluidine Blue or Yariv), calculating the area of the region of interest and the proportion occupied by gum. Both macros are integrated in a toolset, publicly available at https://github.com/SiggRAD/PHIV_ImageJ_Toolsets as “PHIV_MesureAcacia_toolset.ijm”, allowing reproduction of all analyses presented in this study.

Multiphoton images were analyzed using ZEN black software (ZEISS). Specifically, the images were deconvolved using reference spectra acquired from pure Alexa 546, allowing the validation of the secondary antibody signal and confirming the in-situ localization of the JIM4 epitope of arabinogalactan gum. The spectra employed for deconvolution included Alexa 546, as well as signals corresponding to water-soluble tannins and polyphenol-containing cells.

### Statistical analysis

All quantitative data were analyzed using R software (version 4.3.3-x86-64) (R Core Team 2017). Normality and variance homogeneity were checked with Shapiro–Wilk and Levene tests. ANOVA followed by Tukey’s HSD was used for normally distributed data, and Kruskal–Wallis with Bonferroni correction for non-normal data. Results are presented as mean ± SE. Boxplots show the median (line), interquartile range (box), and whiskers (min–max within 1.5× IQR). Statistical significance is indicated by asterisks (*p < 0.05; **p < 0.01; ***p < 0.001).

## Results and discussion

### Results

#### Bark anatomy and histochemistry during the non gummosis period

Whatever the sample analyzed, the bark of *Senegalia senegal* collected on adult trees during the non-gummosis period (October) displays a consistent anatomical organization. On transverse sections stained with toluidine blue, from the cambium to the most superficial part of the bark, 5 distinct anatomical zones can be distinguished: the inner secondary phloem (424 μm), the outer secondary phloem (2379 μm), the pericyclic sclerenchyma (96 μm), the cortical parenchyma (250 μm), and the periderm (312 μm).

**Fig. 1:**
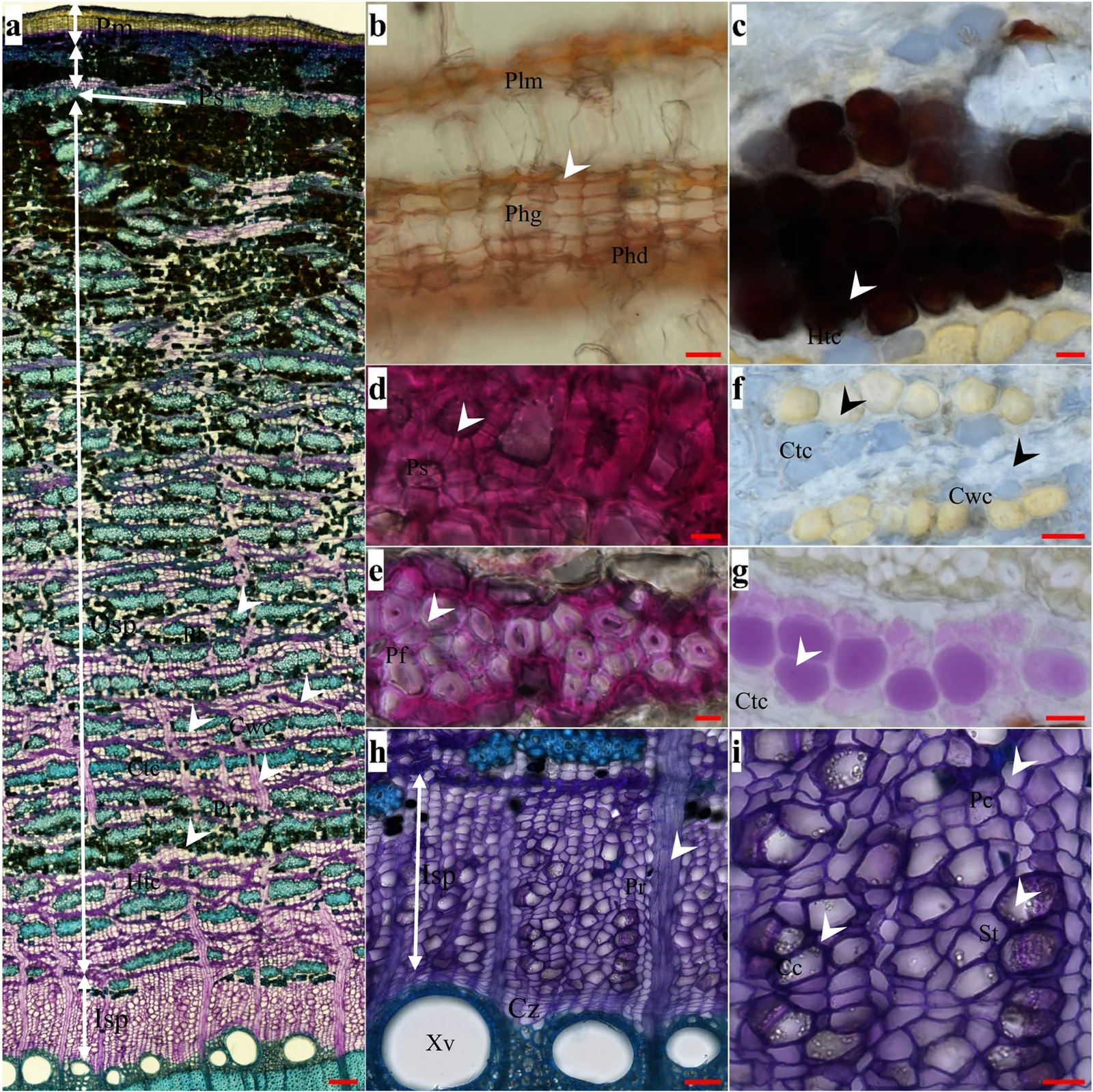
Anatomical organization and histochemistry of the bark of *Senegalia senegal* during the non gummosis period (October) (**a**) General view of the Transversal bark section of *Senegalia senegal* bark (toluidine blue staining). (**b**) Detail of the periderm zone (oil red staining). (**c**, **e**, **f**, **g**) Detail of the outer secondary phloem zone, (**c**) Cluster of hydrolysable tannin cells (arrow) (Stiasny reagents staining), (**e**) Cluster of phloem fibers from outer secondary phloem (phloroglucinol staining), (**g**) Cluster of condensed tannin cells (arrow) (Vanillin-Hcl staining). (**h**, **i**) Detailed view of inner secondary phloem (toluidine blue staining). (**d**) Detail of the Pericyclic sclerenchyma zone (Phloroglucinol staining). *Cc* companion cells, *Cp* Cortical parenchyma, *Ctc* Condensed tannin cells, *Cwc* Cell-walls of collapsed cells, *Cz* Cambial zone, *Htc* Hydrolysable tannin cells, *Isp* Inner secondary phloem, *Osp* Outer secondary phloem, *Pc* Parenchyma cells, *Pf* Phloem fibers, *Phd* phelloderm, *Phg* phellogen, *Plm* Phellem, *Pm* Periderm, *Pr* Phloem rays, *Ps* Pericyclic sclerenchyma, *St* sieve tubes, *Xv* Xylem vessel, Scale bar: a, e = 200 μm; b, c, d, e, f, g, h = 20 μm

The bark appears darker in the outermost areas and gradually becomes lighter towards the inner tissues (Fig. 1a). The periderm (Pm) and cortical parenchyma (Cp) form the outermost layers, followed by the pericyclic sclerenchyma (Ps). Next comes a large zone occupied by the outer secondary phloem (Osp). This is followed by the inner secondary phloem (Isp), which extends between the cambial base (Cz) and the first collapsed cell walls.

The periderm, stained with 0il Red, shows that its cell walls are impregnated with lipid compounds, which appear as red staining (Fig. 1b). Below the pericyclic sclerenchyma (Ps) hydrolysable tannin cells (Htc) are present in the outer secondary phloem (Osp) and grouped in clusters (Fig. 1c) with a thickness of approximately 15.74 μm +/−1.57. They are stained dark reddish-black by the Stiasny + FeCl_3_ reagent, which reveals the presence of catechin-type tannins. This area also contains clusters of pericyclic bast fibers (Pf) (Fig. 1d and e). In addition, condensed tannin cells (Ctc) are observed, which are stained beige by the Stiasny reagent, and clusters of collapsed cell walls (Cwc) are interspersed between them (Fig. 1f). Using vanillin-HCl staining, these cells are stained purple (Fig. 1g).

In the inner secondary phloem (Isp), there are sieve tubes (St), companion cells (Cc), phloem parenchyma cells (Pc), and phloem rays (Fig. 1h and i). These sieve tubes are more or less regularly shaped, with an average thickness of 26.16 +/- 1.85 μm and contain variable numbers of amyloplasts. During this non-gummosis period, the thickness of the radial thickness of the cambial cells in transverse section is approximately 3.82 μm +/- 0.7 and the number of cell layers is about 9 +/- 1.14.

#### Bark anatomical and histochemical changes of the during gummosis period

**Fig. 2:**
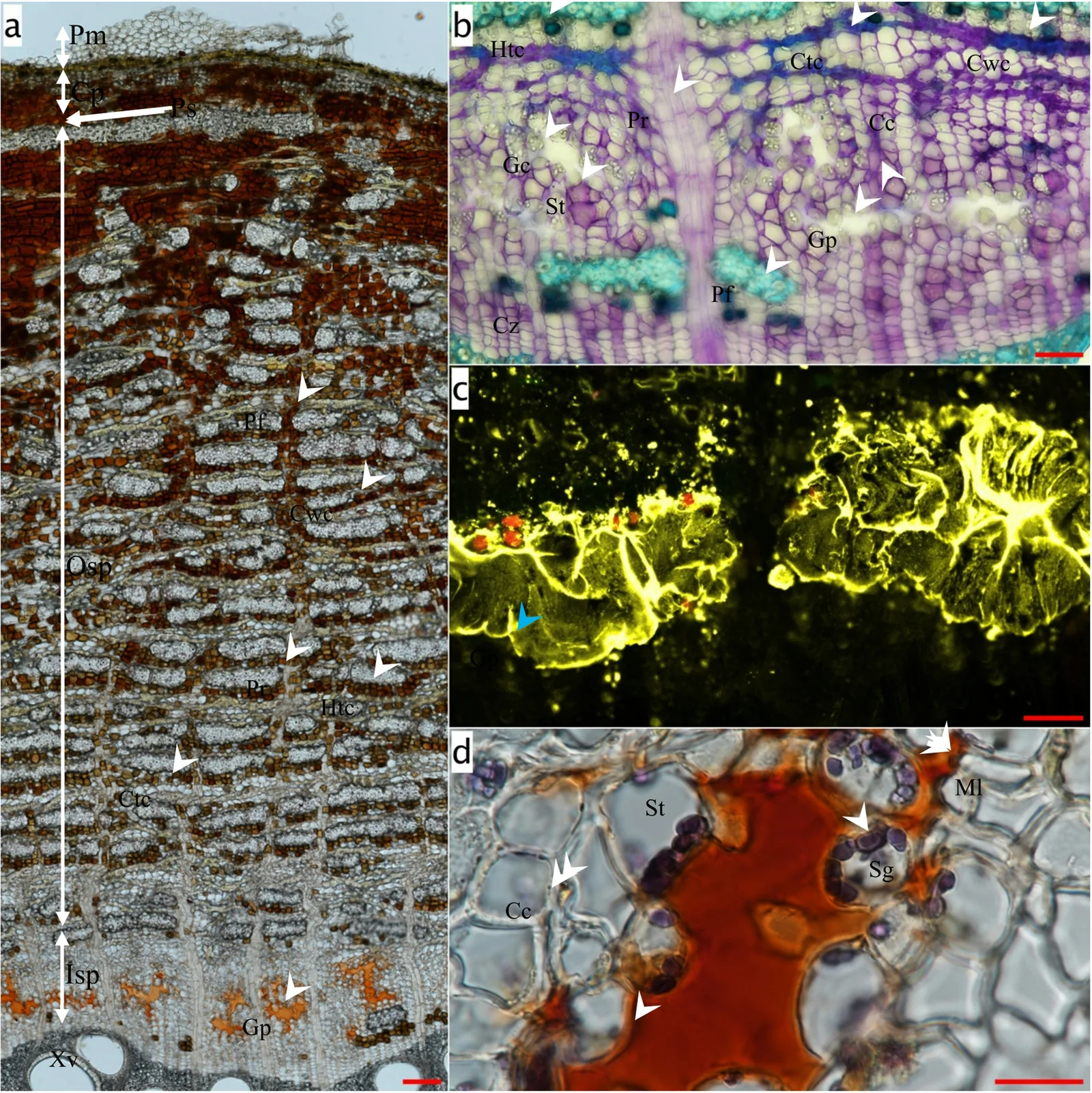
Anatomical organization of the bark of *Senagalia senegal* during the gummosis period (march). (**a**) General view of a Transversal section of *Senegalia senegal* bark (Yariv reagent staining) Cz: Cambial zone. (**b**) Inner secondary phloem with Gum pockets (Yariv reagent staining), (**c**) Gum pockets immunolabeled by JIM4 antibody (yellow signal), (**d**) Gum pockets stained by Yariv reagent (in orange/red) and starch granules stained by Lugol (in black purple), Scale bar: (a) = 100 μm; (b, c) = 50 μm; (d) = 20 μm. Cc: companion cells, *Cp* cortical parenchyma, *Ctc* Condensed tannin cells, *Cwc* Cell-walls of collapsed cells, *Cz* Cambial zone, *Gp* Gum pocket, *Htc* Hydrolysable tannin cells, *Isp* Inner secondary phloem, *Ml* Middle lamella. *Osp* Outer secondary phloem, *Pf* Phloem fibers, *Pm* periderm, *Pr* phloem ray, *Ps* pericyclic sclerenchyma, *Sg* Starch grains, *St* sieve tubes, *Xv* xylem vessel

During the gummosis period, the bark exhibits a histological structure similar to that observed during the non-gummosis period, but with a major difference: the presence of gum pockets within the inner secondary phloem. These pockets are formed in the central region of the inner secondary phloem and are evenly distributed around the circumference of the trunk. It should also be noted that the phloem rays are not affected by this process. Indeed, no gum pockets form within the rays; they develop exclusively between them without altering their structure. They all appear between the rays without affecting them. Toluidine blue-stained sections during the gummosis period also reveals the presence of these lacunae stained blue, indicating their affinity for this dye (Fig. 2a). Around the gum pockets, three main cell types can be observed: sieve tubes (St), companion cells (Cc) and phloem parenchyma cells (Pc).

Staining with Yariv reagent (specific for AGPs) shows that these pockets strongly accumulate AGPs (Fig. 2a) and combined Yariv’s reagent and Lugol staining reveals that the cells lining the gum pockets contain numerous starch granules (Fig. 2b). By immunolocalization using the antibody JIM 4, known to detect the AGP epitope of arabic gum, we were able to show that this epitope.

accumulates strongly within the pockets located in the inner secondary phloem during gummosis (Fig. 2c). Taken together, these histo-immunochemical analyses of the pockets formed during the gummosis period correspond to sites of gum Arabic accumulation, thus justifying the term “gum pockets”.

Furthermore, Lugol iodine staining combined with Yariv reagent during the gummosis period highlights starch reserves (bluish black starch granules) mainly within the cells bordering the pockets (Fig. 2d).

During gummosis period, the thickness of the cambial cells in the radial transverse section direction was around 9.49 μm +/- 1.02 and the number of cambial cells lay around 4, compared to 3.82 μm and about 9 cell layers during the non-gummosis period.

**Fig. 3:**
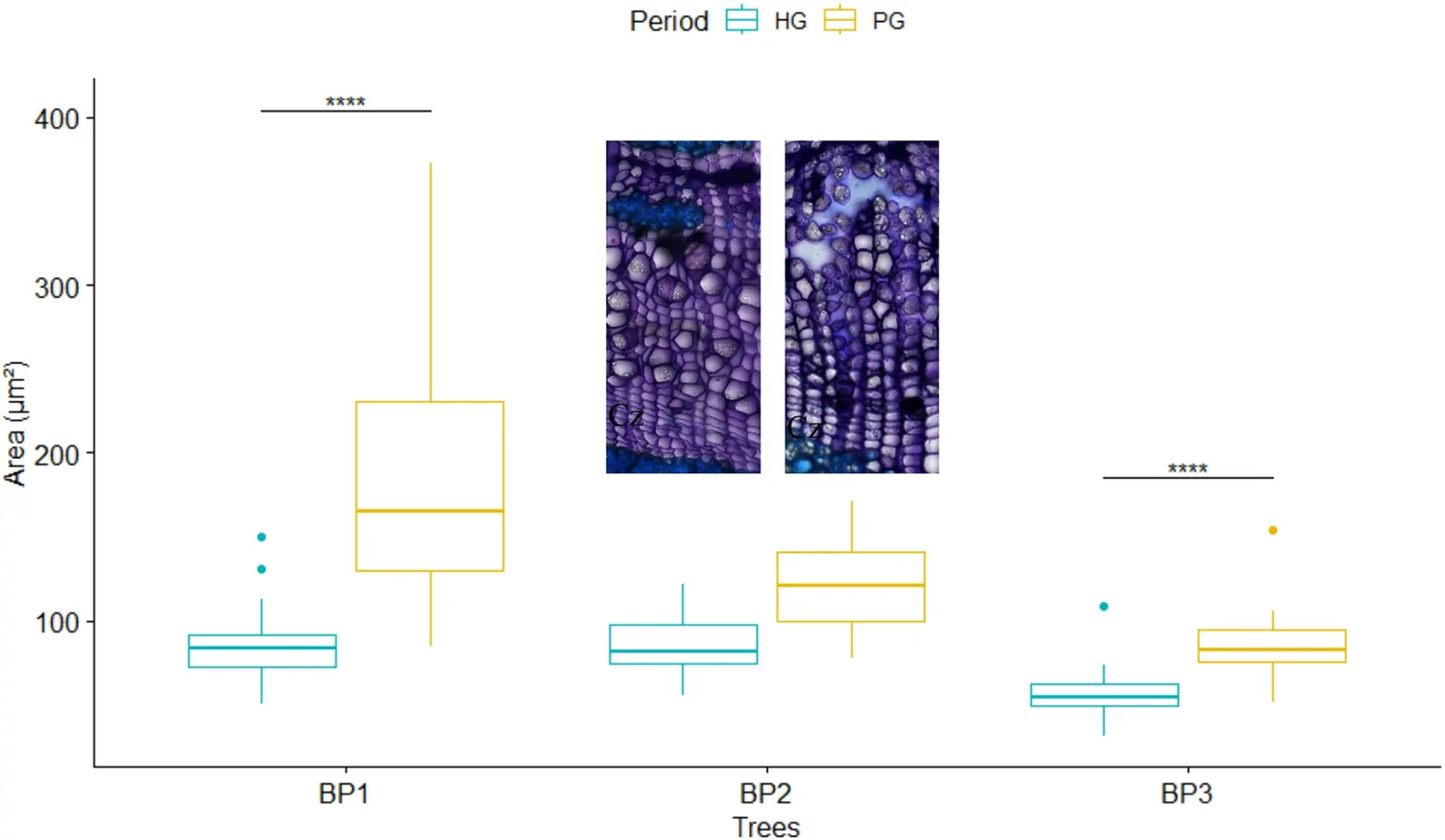
Comparative study of cambium cell area during the gummosis and the non gummosis period: cambium cell area increase during the gummosis period (µm^2^). *Cz* Cambial zone, *BP* Bader Producer, *HG* non gummosis period, *PG* gummosis period, ****= highly significant difference

The analysis of the variation in t cambial cells size showed that their area differs depending on whether the tees are in the gummosis period or not. This analysis indicates that cambial cell area **is significantly** higher (F value = 68.66 and *p* = 2.56E-13) during gummosis period than during non-gummosis period (Fig. 3; (Fig. 3). For the 3 sample trees, values ranged between 50 and 100 µm^2^ during the non-gummosis period, whereas they ranged between 100 and 240 µm^2^ during the gummosis period.

In addition to the presence of gum pockets in the inner secondary phloem, the gummosis period is characterized by changes in the cambial zone. These changes are reflected in an increase in the number of cell layers (6–8 on average, compared to 3–4 during the gum production period) and a modification of cambial cell shape.

**Fig. 4:**
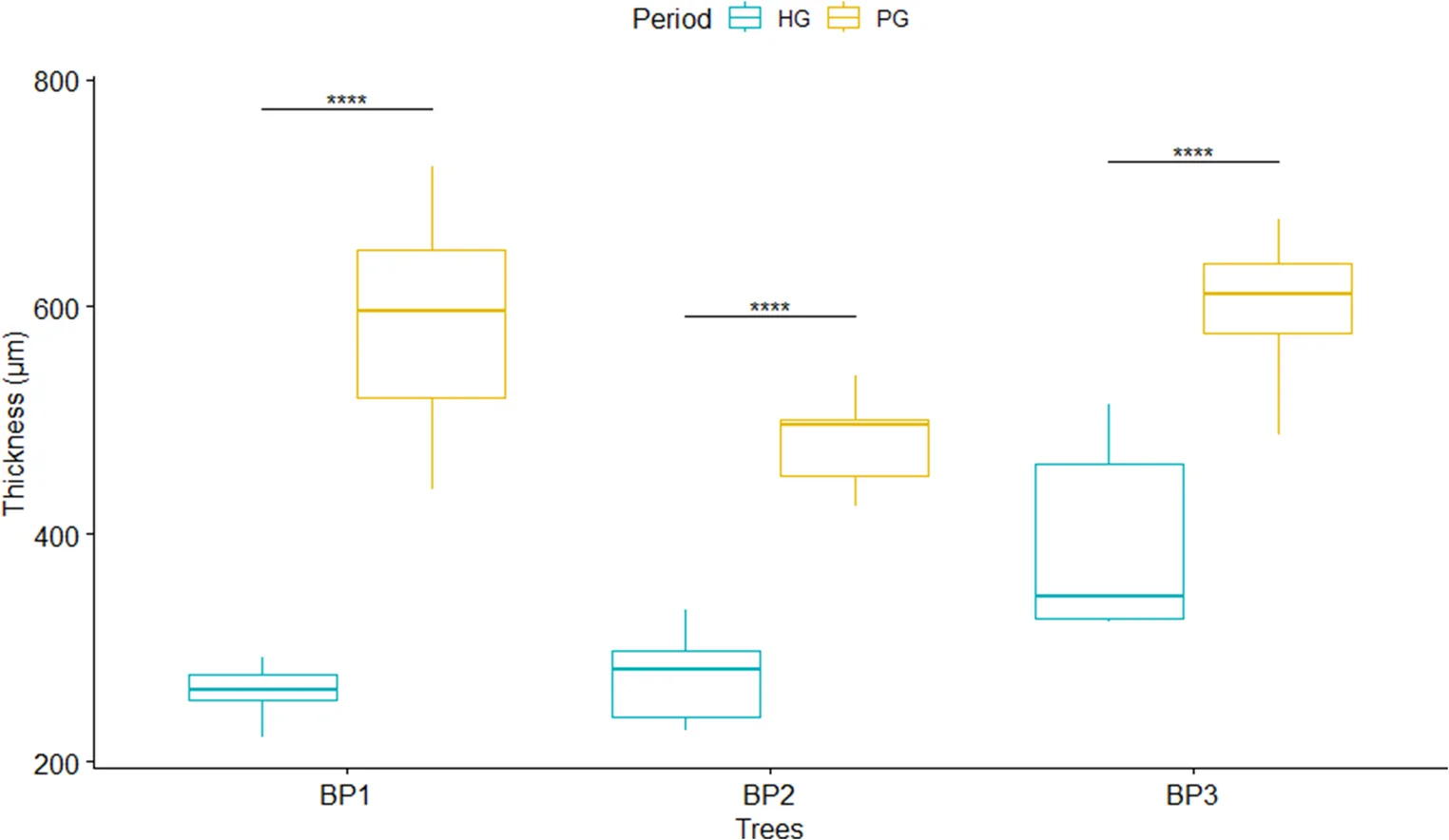
Comparative study of inner secondary phloem thickness during the gummosis and non gummosis period (µm). **Genotype: ***BP* Bader Producer, *HG* non gummosis period, *PG* gummosis period, ****= highly significant difference

The evolution of cambial zone thickness was also analyzed between gummosis and non-gummosis periods. The results show that this parameter is significantly higher during the gummosis period than during the non-gummosis period (F value = 5.71 and *p* = 0.006; Fig. 4). The averages of thickness of this zone ranges from 261.6 μm to 393.16 μm during the non-gummosis period and from 420.94 μm to 604.89 μm during the gummosis period, depending on the trees.

#### Ontogenesis of gum pockets

**Fig. 5:**
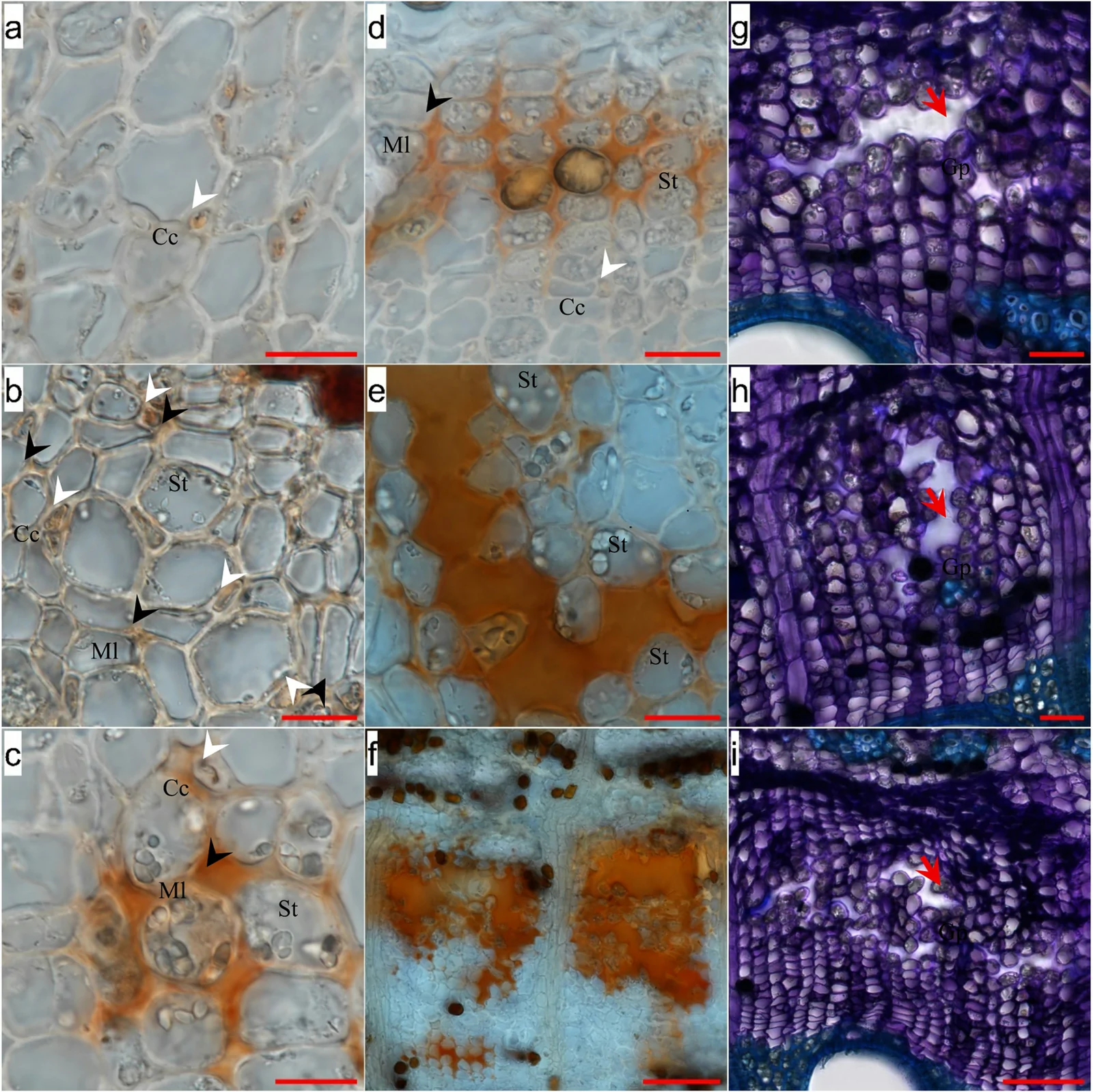
Histochemical events leading to the genesis of gum arabic pocket in the inner secondary phloem, during the gummosis period. Images a to f (Yariv reagent staining) illustrate the progressive stages of gum pockets formation. First, Yariv signal is detected in companion cells nucleus (image **a**), then it accumulates in the intercellular spaces (image **b**). The accumulation of AGP begins in enlarged intercellular spaces (image **c**) and extends to the surrounding cell walls (image **d**). The cell separation process then expands (image e and progressively occupies in the inter-ray spaces (image **f**). Images **g** to **i** (toluidine blue saining) illustrate that the schizogenous gum pocket formation can occur in tangential (image **g**) or radial (image **h**) directions and may originate from multiple initiation sites (image **i**). Scale bar: (a, b, c, d, e) = 20 μm; (f, g, h, i) = 100 μm

To describe the kinetics of formation of g pockets, a staggered sampling was carried out from October to March. Sections were stained by Yariv reagent. The earliest event observed is an increase in Yariv signal intensity in the companion cells of the inner secondary phloem zone (Fig. 5a, March). Subsequently, an accumulation of the Yariv signal is observed in the intercellular meatus of the cell walls which enlarges as AGPs accumulate, extending from the middle lamella to the entire wall (Fig. 5b). The walls thicken and a cell separation phenomenon gradually develops, leading to the formation of intercellular spaces that give rise to pocket openings (Fig. 5g).

The separation occurs in both the radial (Fig. 5g-e) and tangential/lateral (Fig. 5e) directions, while sparing the phloem rays. This mechanism often starts with multiple initiation sites (Fig. 5i-f), with 3 to 4 initiation sites forming between two phloem rays which then expand in all directions and eventually fuse to form a single cavity occupying almost the entire space between two adjacent rays.

AGPs progressively accumulate in the apoplasm through the middle lamella from companion cells (Fig. 5b). The signal becomes more intense in the middle lamella as AGPs accumulate. AGPs continue through an expansion in the neighboring cells (Fig. 5d). This is followed by a schizogenous intercellular separation, where openings form progressively between cell walls at the middle lamella level (Fig. 5e). This opening is progressively extended and widened.

Complementarity is observed on both sides of the opening, confirming schizogenesis rather than lysogenesis as a mechanism of cavity formation (Fig. 5e). The opening enlarges to form a large lacuna, the gum pocket (Fig. 5f). Several intermediate stages are observed during the formation of gum pockets (Fig. 5g-i), with the phenomenon of cell spacing occurring both radially (Fig. 5g) and tangentially (Fig. 5h), always sparing the phloem rays. Multiple initiation sites (3–4 between two rays) evolve in all directions and fuse to form a single cavity that expands to occupy almost all the space between two adjacent secondary phloem rays (Fig. 5i).

**Fig. 6:**
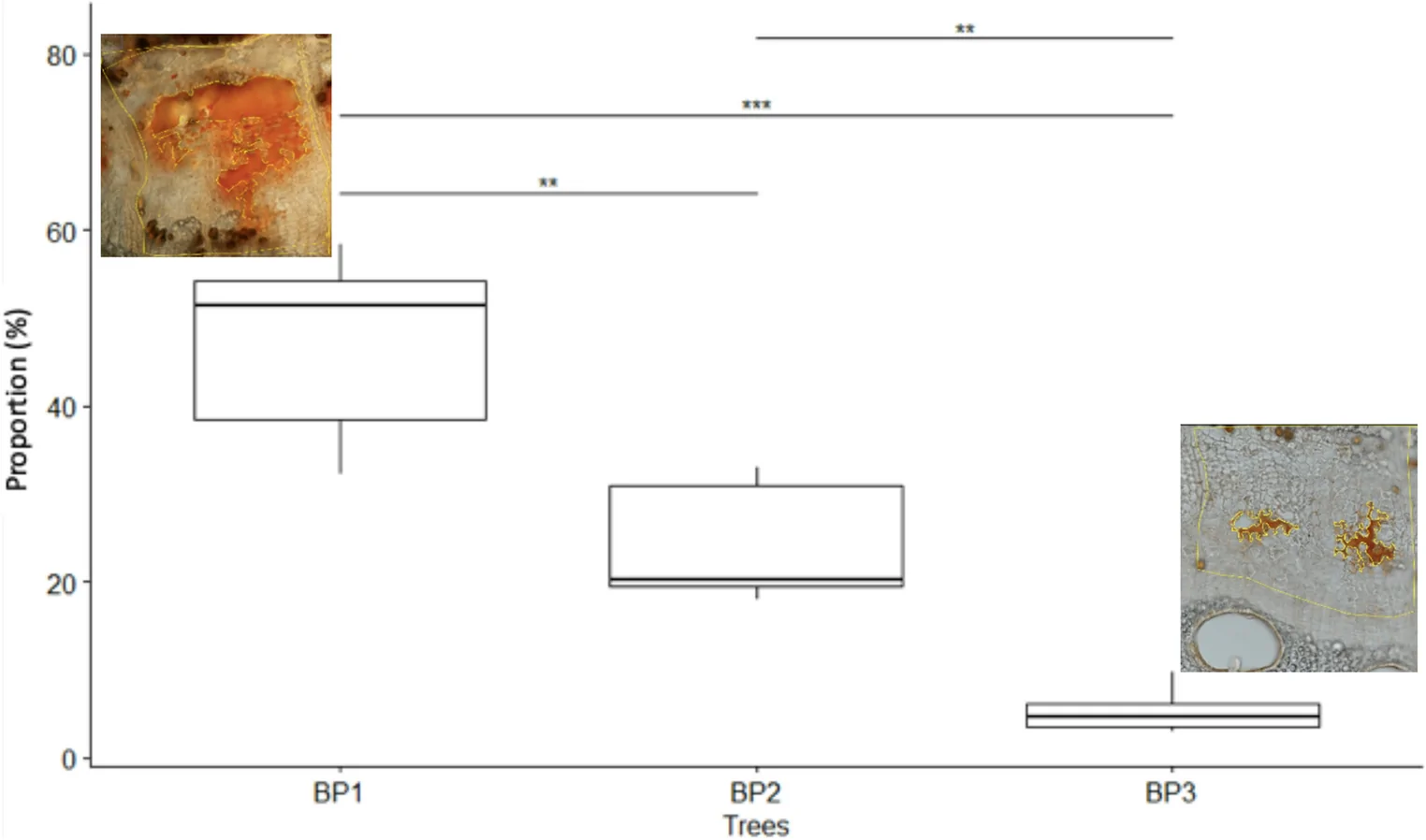
Proportion (%) of the inner secondary phloem occupied by gum pockets. Transversal bark sections were stained by Yariv reagent. Correlation between gum yield and the proportion of the inner secondary phloem occupied by gum pockets

**Fig. 7:**
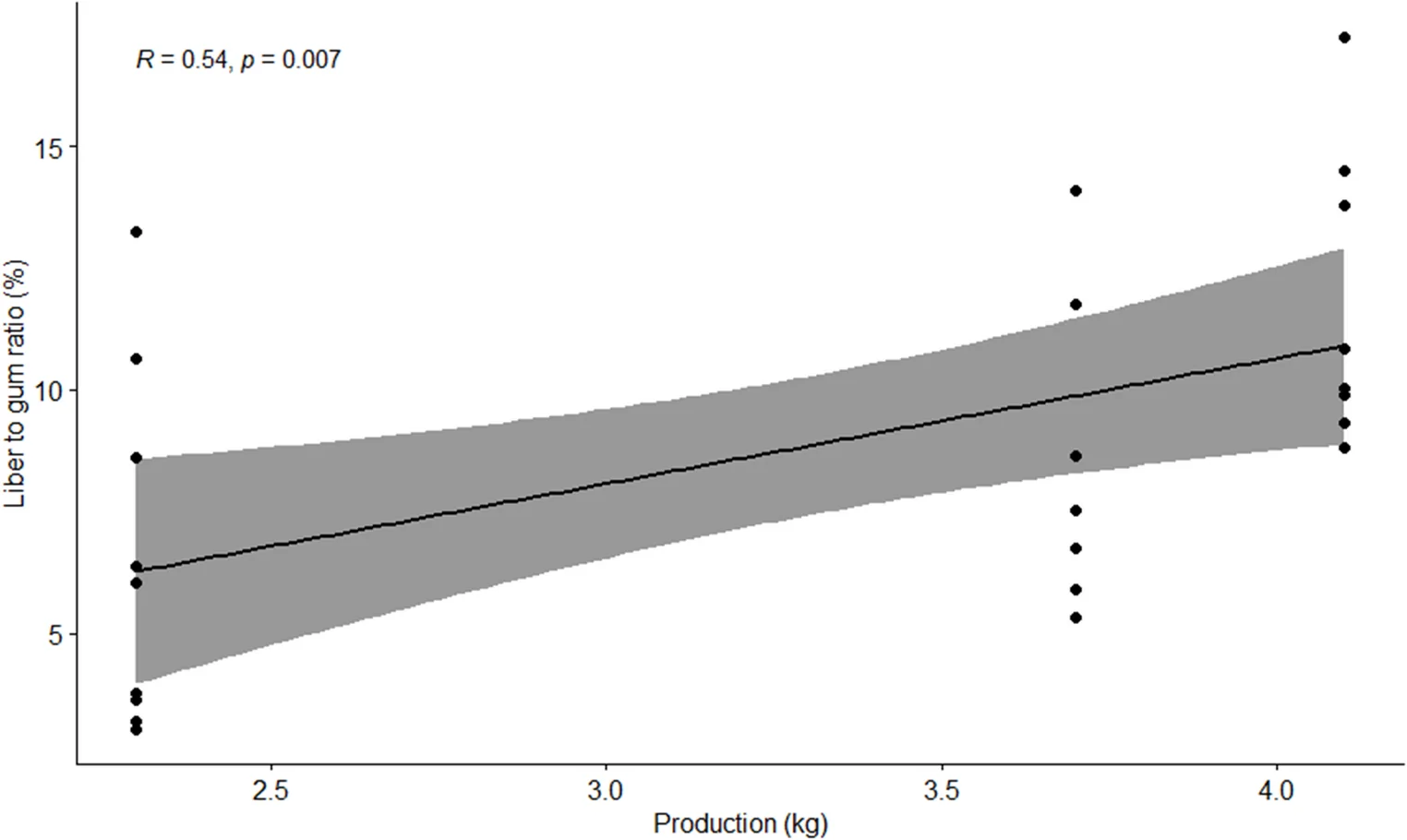
Correlation between gum production and the proportion of the inner secondary phloem occupied by gum pockets

We measured the surface area of the gum pockets and calculated the ratio of this area relative to the inner secondary phloem located between two adjacent phloem t rays. The analysis shows that the proportion of the inner secondary phloem occupied by gum pockets (stained with Yariv reagent) varies among trees sampled on the same day (Fig. 6). The ratio varied considerably from 39 to 55% in the tree BP1 to 5–8% in the tree BP3 (5% to 8%). A comparison between gum yield and the production of gum pockets among different tress shows that the amount of gum harvested is positively correlated with the area occupied by gum pockets in the inner secondary phloem (correlation coefficient *R* = 0.54, *p* = 0.007) (Fig. 7).

#### Evolution of gum pockets during the transition from the gummosis period to the non-gummosis period

**Fig. 8:**
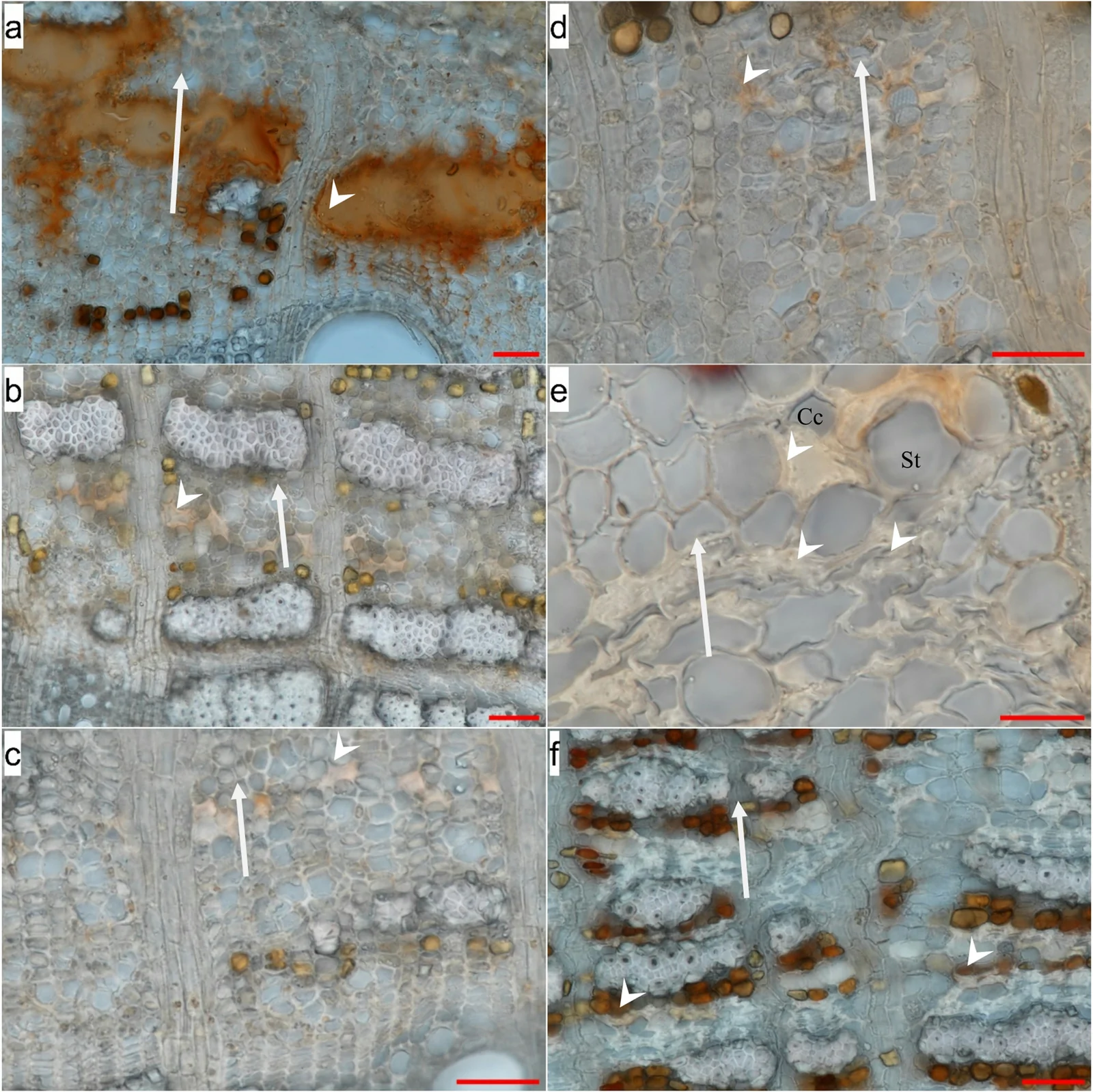
Evolution of gum pockets during the transition from the production to the non-production period: progressive resorption. Transversal bark sections stained with Yariv reagent staining). At the late stage of gummosis, gum pockets are well developed (image **a**). They then begin to shrink, but remain close to the cambial zone (image **b**). Traces of the old gum pockets progressively move away from the cambium (image **c**) and are located very farther from the cambium towards the end of the inner secondary phloem (image **d**), Closure of gum pockets occurs concurrently collapse of the sieve tubes and the formation of clusters of collapsed cell walls (image **e**). Finally, residual AGP signals from the old pockets are observed within the clusters of collapsed walls in the outer secondary phloem (image **f**). cell. White arrow: gum pockets resorption direction, *St* sieve tubes, *Cc* companion cells. Scale bar: (a, b, c, d, f) = 50 μm; (e) = 20 μm

We were interested in the fate of the gum pockets after the production period, as they are no longer visible outside this period. To address his question, sampling was conducted between March and April to monitor the evolution of gum pockets during the transition from the production period to the non-production period. Like their formation, gum pockets resorption is a gradual process that occurs over several months (October to March). Several intermediate stages were identified, ranging from fully differentiated gum pockets to their complete resorption. Yariv reagent staining was used to characterize these stages (Fig. 8). During the gumming period, the differentiated gum pocket occupies almost all the spaces between two adjacent rays of the inner secondary phloem (Fig. 8a). At this stage, it the inner secondary phloem is devoid of differentiated pericyclic phloem fibers. The resorption of the gum pockets starts with a decrease of the intensity of the Yariv reagent staining and an anisotropic shrinkage of the pockets, primarily in the direction of their width (perpendicular to the cambial cell layers). (Fig. 8b). The onset of gum pockets resorption is accompanied by the concomitant differentiation of the first pericyclic fibers within the inner secondary phloem.

Despite the neo-formation of the fibers below, traces still remain close to the cambial zone. As new inner secondary phloem tissues are generated, the pockets progressively move away from the cambium (Fig. 8c). At this stage, the labelling signal weakens and disappears between the gum pockets’ marks. Then the signal continues to move further away and becomes even weaker (Fig. 8d). A progressive loss of structure is observed in the sieve tubes surrounding the old gum pockets. Later, these sieve tubes collapse, forming new clusters of collapsed walls in the intermediate zone between the inner secondary and outer secondary phloem (Fig. 8e). Finally, a faint residual Yariv signal remains within the clusters of collapsed walls in the outer secondary phloem (Fig. 8f).

#### Immunohistochemical characterization of clusters of collapsed cell walls

**Fig. 9:**
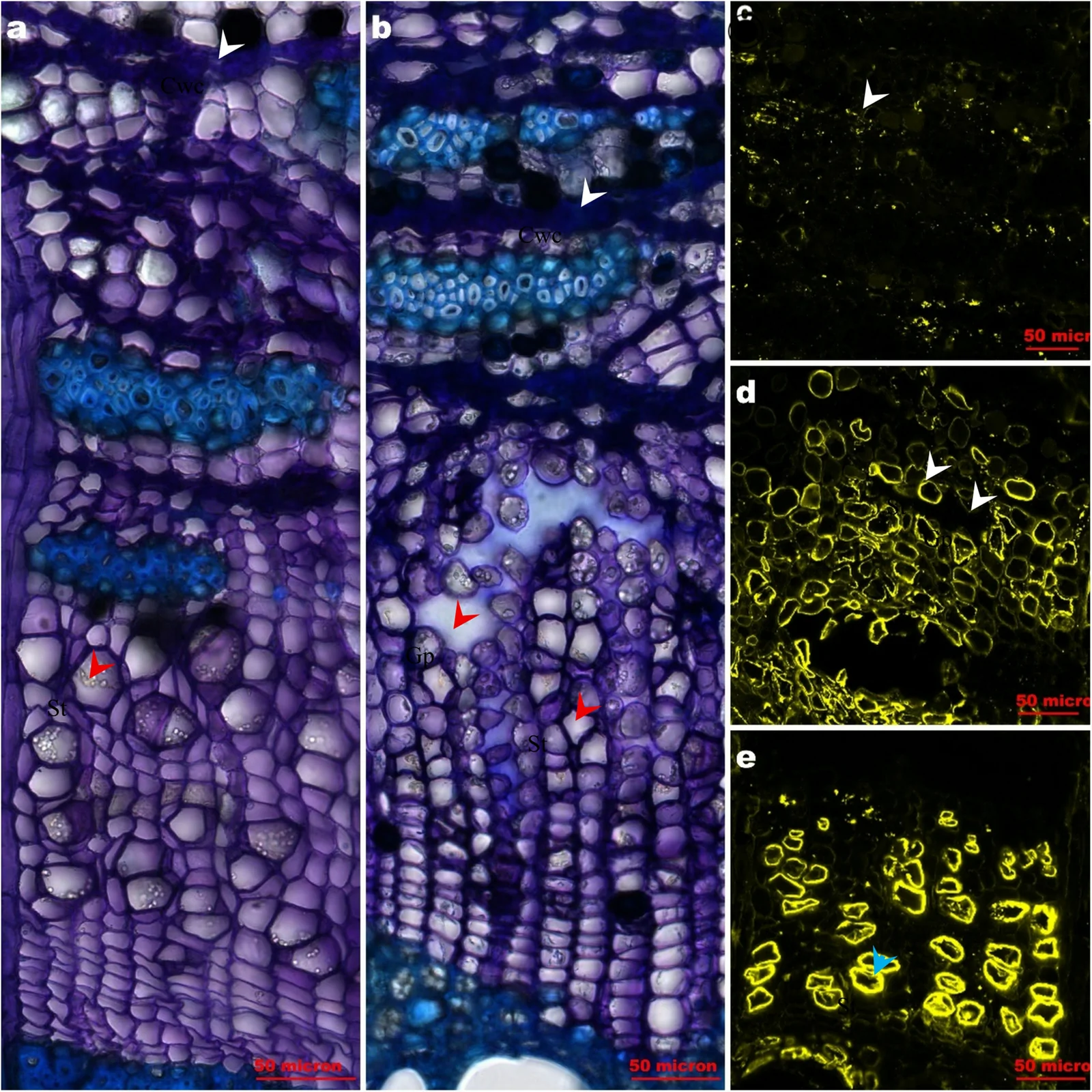
Clusters of collapsed cell walls and their probable origin.: Transverse sections of the inner secondary phloem stained with toluidine blue: non-gummosis period (**a**) and gummosis period (**b**). Imunohistochemical characterization of the clusters using the LM26 antibody (β-(1,4)-galactans, labeling sieve tube cell walls in yellow: collapsed cell walls (**c**), sieve tube surrounding a gum pocket (**d**) and sieve tube near the cambium (**e**); Cwc: Cell-walls of collapsed cells, Gp: Gum pocket. Stw: sieve tubes walls

The clusters of collapsed cell walls form simultaneously with the resorption and closure of gum pockets and the behavior of the sieve tubes at the onset of cluster formation prompted an investigation into a possible link between these events. These clusters were visualized using s Toluidine blue staining (Fig. 9a and b). They are located in the outer secondary phloem, forming continuous, blue-stained structures. Their thickness ranges from approximately 9 to 34 μm. Immunolocalization using LM26 antibody (β-(1,4)-galactans epitope, labelling sieve tube cell wall) was then applied to investigate this link. It was observed that the antibody signal specifically labels the wall of sieve tubes in the intact inner secondary phloem during the non-gummosis period (Fig. 9e) or those surrounding pockets in the gummosis period (Fig. 9d). In the outer secondary phloem, the same signal was detected in the clusters of collapsed cell walls (Fig. 9c). It should be noted that the labeled elements in the inner secondary phloem correspond to the regions where the gum pockets form during gummosis.

### Discussion

Our study aimed to better understand the histological and histochemical mechanisms underlying gum arabic formation in *Senegalia senegal*, an emblematic species of the Sahelian region. Combining histology, histochemistry, and immunolocalization, we provide new insights into bark organization, the genesis of gum pockets, their chemical composition, seasonal dynamics, and their potential role in cambial reactivation during the transition from the dry to the wet season.

### A radially organized bark, typical of Fabaceae, with functional specificities

The bark of *Senegalia senegal* exhibits a radial organization characteristic of that described in other arborescent Fabaceae, particularly species within the Caesalpinioideae and Mimosaceae subfamilies (León-H 2008; Carmo et al. 2016a; Mota et al. 2017). It is stratified into seven well-differentiated zones from the inside outward: the vascular cambium, the inner secondary (often referred to as functional) phloem, the outer secondary phloem (non-functional) lacking sieve tubes, a concentric pericyclic sclerenchyma, a phenolic-rich cortical parenchyma, and the periderm.

Several anatomical features observed in *Senegalia senegal* are shared with other Fabaceae, such as the presence of multiseriate phloem rays that dilate progressively in the outer secondary phloem toward the bark periphery as a result of tangential stretching and anticlinal cell divisions. The dilatation phenomenon is known to enable the bark to accommodate the radial growth through parenchyma cell division and cell expansion (Angyalossy et al. 2016).

The outer secondary phloem of *Senegalia senegal* exhibits a highly organized structure, characterized by a repetitive pattern of four main cell types arranged in clusters: phloem fibers, aggregates of collapsed cell walls, cells containing condensed tannins (often referred to as secretory cells; Angyalossy et al. 2016), parenchyma cells, and cells rich in hydrolysable tannins. While clusters of tannin-containing cells are commonly reported in the bark of Fabaceae, our use of specific histochemical stains allowed us to distinguish of two distinct types of tannin cells in *S. senegal*: (i) cells containing hydrolysable tannins, which stain dark red with Stiasny’s reagent, and (ii) cells containing condensed tannins, which appear beige with Stiasny’s reagent and violet with vanillin-HCl. This histochemical differentiation highlights the remarkable diversity of tannin-producing cell types in the bark of *Senegalia senegal*, which may be functionally associated with the species deterrent properties against herbivores in Sahelian ecosystems (Balises 2020).

Unlike other Fabaceae, where a gradual transition is typically observed between intact sieve tubes, collapsed sieve tubes, and phloem fiber masses, this transition appears abrupt in *Senegalia senegal*. Only the immunolocalization of the LM26 epitope, specific to phloem tissues (Torode et al. 2018), allowed us to demonstrate that these cell wall aggregates originate exclusively from collapsed sieve tubes.

### The inner secondary phloem: an exclusive and spatially controlled site of gum pocket formation

Our observations demonstrate that the inner secondary phloem is the primary site where gum pockets originate and develop in *Senegalia senegal*. Our histochemical analyses, carried out during the peak period of gum exudation, combined Yariv reagent staining with immunolabeling using the JIM4 antibody, which specifically recognizes arabinogalactan proteins (AGPs) characteristic of gum arabic. For the first time, this approach provides direct evidence of the chemical identity of gum pocket contents and allows precise in situ localization of gum formation sites.

In contrast to the findings of Lutz (1895), we did not observe any lacunar formation within the bark pericycle. Our observations clearly indicate that gum pocket formation is a localized phenomenon restricted to the inner secondary phloem, specifically between the cambial zone and the first wall aggregates of collapsed cell walls. Importantly, gum formation systematically avoids the phloem rays, contrary to earlier reports by Ghosh and Purkayastha (1962) and Mouret (1987). In addition, the study identified the cell types located around the gum pockets in the inner secondary phloem including sieve tubes, companion cells and phloem parenchyma cells. Our results suggest a finely tuned and conserved spatial control of gum pocket formation within the inner secondary phloem of *Senegalia senegal*.

Furthermore, preliminary morphometric analyses suggest a strong correlation (R² = 0.87) between gum yield and the ratio of gum pockets area to inner secondary phloem area (between adjacent rays). If validated on a larger set of trees, this ratio could provide a valuable anatomical proxy for selecting high-yielding individuals, opening new perspectives for breeding and domestication programs targeting gum production.

### Spatiotemporal sequence of cellular events leading to gum pocket formation

Time-staggered sampling combined with histochemical characterization allowed us to propose a detailed sequence of cellular events leading to the formation of gum-accumulating pockets. This sequence can be summarized as follows:

1. Increased biosynthesis of arabinogalactan-proteins (AGPs) and accumulation of the JIM4 epitope in the cytoplasm of companion cells;
2. Accumulation of AGPs and JIM4 labeling at the cell wall corners of companion cells, parenchyma cells, and sieve tubes, with progressive spread into the middle lamella;
3. Cell wall thickening of cell walls accompanied by stronger Yariv staining and increased accumulation of the JIM4 epitope;
4. Formation of intercellular spaces, marking the onset of gum pocket development.

Initially confined to the companion cells of the inner secondary phloem, the process extends to adjacent sieve tubes and hydrolysable tannin cells. Multiple initiation sites were observed within the inner secondary phloem. The resulting intercellular spaces eventually coalesce to form a single gum pocket between two adjacent phloem rays. As previously established in our study, once initiated, gum pockets expand radially and/or tangentially to form large reservoirs through the progressive fusion of these initiation sites.

### Companion cells: key initiators of gum formation and schizogenous origin of gum pockets

Our study provides the first direct evidence that companion cells act as the primary initiators of gum formation and demonstrates that gum pocket development in *Senegalia senegal* is driven by a *schizogenous* mechanism, i.e., a controlled separation of cells, rather than by lysigenous degradation, as previously hypothesized by Ghosh and Purkayastha (1962) and later supported by Mouret and Vassal (1991), although without histochemical validation. While some isolated cells may be trapped within the developing gum pockets, we did not observe any evidence of cell lysis. Cells located either within the pockets or along their periphery *retained their structural integrity and cytoplasmic contents. In contrast to earlier reports by* Mouret (1987*)*,* we did not* observe any breakdown of sieve tubes, tannin cells, fibers, or ray cells during gum pocket formation. *Schizogenous separation is a well-documented developmental mechanism in plants and plays a role in various biological processes*,* such as abscission.* In this context, our findings suggest that gum pocket formation in *Senegalia senegal* occurs through a spatially controlled schizogenous process.

### Seasonal dynamics of gum pocket resorption: fate of non-exuded gum

One particularly novel aspect of our study is the observation of gum pocket resorption following the active gummosis period. This process occurs within a few weeks after the end of the dry season, ultimately leading to the complete disappearance of the gum pockets. The decrease in both Yariv staining and JIM4 labeling in trees that did not exude gum suggests metabolic reabsorption of gum components, a phenomenon not previously documented. This raises an important question: what becomes of the non-exuded gum? We hypothesize that it may be metabolically reutilized by the tree as a source of carbon and nitrogen, particularly to support cambial reactivation and the formation of new phloem tissues. Supporting this, we observed cambial activity and new sieve tube formation in samples collected in March, prior to budburst and leaf emergence, coinciding with the end of the dry season.

This early onset of phloem regeneration suggests the existence of an internal carbon source reserve. Consistent with previous findings by Joseleau (1983) and Joseleau and Ullmann (1985), our observations indicate a significant reduction in starch reserves in the rays adjacent to the cambium and gum pockets, along with increased amylase activity during peak gummosis.

We therefore propose that the resorbed gum, rich in arabinogalactans (polysaccharides) and proteins, may serve as a transient metabolic reserve. This physiological strategy likely represents an adaptive to seasonal constraints in the Sahelian environment, allowing *Senegalia senegal* to anticipate foliar development by ensuring early formation of new functional phloem tissues, including sieve tubes and companion cells.

Overall, our results suggest that gum arabic acts not only as a defense-related exudate but may also function as an internal reserve, mobilized during the critical transition between the dry and rainy seasons.

## Conclusion

Despite its ecological and socio-economic importance, the *Senegalia senegal* species has been little studied, particularly regarding the biosynthesis mechanisms of gum, a substance highly valued by industry by the industrial sector. The present research allowed us to better characterize the tree’s bark histologically but also to identify the sites of gum accumulation using reliable histochemical tools such as Yariv and JIM4, which are specific to PFAs, the essential components of this product. In fact, gum pockets develop exclusively in the inner secondary phloem. Moreover, we were able to describe the kinetics of gum pocket formation as a schizogenous, rather than lysigenous, process, as suggested by previous studies. Gum synthesis is thought to be associated with companion cells, as evidenced by Yariv labelling of their nuclei. Additionally, we described the resorption mechanism of gum pockets was described, leading to the formation of clusters of collapsed walls derived from sieve tubes mechanically compressed by the pressure of newly generated cambial tissue. This observation provides further evidence that the mechanism does not lead to total lysis of the cells associated with or near the site of synthesis.

## Data Availability

Data will be made available on reasonable request.

## References

[CR1] Acosta CCD, Dias AA, Rosa TLSA, Batista-Silva LR, Rosa PS, Toledo-Pinto TG, Costa F, Lara FA, Rodrigues LS, Mattos KA, Sarno EN, Bozza PT, Guilhot C, Berrêdo-Pinho M, Pessolani MCV (2018) PGL I expression in live bacteria allows activation of a CD206/PPARγ cross-talk that may contribute to successful *Mycobacterium leprae* colonization of peripheral nerves. PLoS Pathog 14(7):e1007151. 10.1371/journal.ppat.1007151 29979790 10.1371/journal.ppat.1007151 PMC6056075

[CR2] Angyalossy V, Pace MR, Evert RF, Marcati CR, Oskolski AA, Terrazas T, Kotina E, Lens F, Mazzoni-Viveiros SC, Angeles G, Machado SR, Crivellaro A, Rao KS, Junikka L, Nikolaeva N, Baas P (2016) IAWA list of microscopic bark features. IAWA J 37(4):517–615

[CR3] Asgar A (2024) La gomme arabique issue des acacias du Sahel africain est utilisée dans des centaines de produits: ce qu’il faut savoir. Magazine The Conversation - Centre d’excellence pour la biotechnologie post-récolte (CEPB) de l’université de Nottingham en Malaisie. https://theconversation.com/la-gomme-arabique-issue-des-acacias-du-sahel-africain-est-utilisee-dans-des-centaines-de-produits-ce-quil-faut-savoir-229051

[CR35] Atgié M (2018) Composition and structure of gum Arabic in solution and at oil-water interfaces (Doctoral dissertation, Institut National Polytechnique de Toulouse-INPT). https://doi.or/10.70675/aa85a437z9622z41f2zbcacz52ff9656b525

[CR34] Buffard-Morel J, Verdeil JL, Pannetier C (1992) Somatic embryogenesis of the coconut palm (Cocos nucifera L.) from leaf explants: histological study. Can J Bot 70(4):735–741. 10.1139/b92-094

[CR4] Carmo JF, Miranda I, Quilhó T, Sousa VB, Carmo FH, Latorraca JV, Pereira H (2016a) Chemical and structural characterization of the bark of *Albizia niopoides* trees from the Amazon. Wood Sci Technol 50(4):Article 4. 10.1007/s00226-016-0807-3

[CR7] Desfemmes C (2026) C’est quoi la gommose ? Actualisé le 13 février 2026. https://www.gerbeaud.com/reponses-experts/gommose,29.html

[CR6] Des plantes intelligentes? L’acacia (2020) Balises, le magazine de la Bibliothèque publique d’information (Bpi). https://balises.bpi.fr/des-plantes-intelligentes-lacacia/

[CR8] Dione M (1996) Recherches expérimentales sur le gommier *Acacia senegal* dans le Ferlo sénégalais. Thèse de doctorat. Université Paul Sabatier de Toulouse, France p 150

[CR9] FAO (1996) Rôle des acacias dans l’économie rurale des régions sèches d’Afrique et du Proche-Orient. CHAIER FAO CONSERVATION. M-32 ISBN 92-5-203651-2

[CR10] Ghosh SS, Purkayastha SK (1962) Anatomical studies of wood and bark of *Acacia senegal* Willd. trees with special reference to gum exudation. Indian Forester 88:92–99

[CR11] Haida S, Bakkouche K, Kribii AR, Kribii A (2021) Chemical composition of essential oil, phenolic compounds content, and antioxidant activity of *Cistus monspeliensis* from Northern Morocco. Biochem Res Int 2021:6669877. 10.1155/2021/6669877 34917418 10.1155/2021/6669877 PMC8670979

[CR12] Hinchman RR (1973) A permanent iodine stain-mountant combination for starch in plant tissues. Stain Technol 48(6):344–346. 10.3109/10520297309116653 4129038 10.3109/10520297309116653

[CR13] Joseleau JP (1983) Sur les hypothèses de l’origine biochimique de la gomme. Acquisitions récentes dans les domaines des hydrocolloïdes végétaux naturels. P. U. Aix-Marseille, pp 19–29

[CR14] Joseleau JP, Ullmann G (1985) A relation between starch metabolism and the synthesis of gum arabic. Bull Int Group Study Mimosoideae 13:46–54

[CR15] Kitazawa K, Tryfona T, Yoshimi Y, Hayashi Y, Kawauchi S, Antonov L, Tanaka H, Takahashi T, Kaneko S, Dupree P, Tsumuraya Y, Kotake T (2013) β-Galactosyl Yariv Reagent binds to the β-1,3-Galactan of arabinogalactan proteins. Plant Physiol 161(3):1117–1126. 10.1104/pp.112.211722 23296690 10.1104/pp.112.211722 PMC3585584

[CR16] Labat J-B (1728) *Nouvelle relation de l’Afrique occidentale, contenant une description exacte du Sénégal et des pays situés entre le Cap-Blanc et la rivière de Sierra Leone, jusqu’à plus de 300 lieux en avant dans les terres, l’histoire naturelle de ces pays, les différentes nations qui y sont répandues, leurs religions & leurs mœurs, avec l’état ancien et présent des compagnies qui y font le commerce…* (Paris: T. Le Gras)

[CR17] León-H WJ (2008) Anatomía de madera en 31 especies de la subfamilia Mimosoideae (Leguminosae) en Venezuela. Colomb For 11:113–136

[CR18] Lutz LC (1895) Contribution à l’étude chimique et botanique des gommes. Thèse Pharmacie, Paris, 92p., 3 pl

[CR19] Mallet B, Besse F, Gautier D, Muller D, Bouba N, et (2003) Quelles perspectives pour les gommiers en zone de savanes d’Afrique centrale ? Savanes africaines: des espaces en mutation, des acteurs face à de nouveaux défis. Garoua, Cameroun, p 11

[CR20] Mota GS, Sartori CJ, Miranda I, Quilhó T, Mori FA, Pereira H (2017) Bark anatomy, chemical composition and ethanol-water extract composition of *Anadenanthera peregrina* and *Anadenanthera colubrina*. PLoS One. 10.1371/journal.pone.0189263 29281656 10.1371/journal.pone.0189263 PMC5744935

[CR21] Mouret M (1987) Les acacias gommiers: recherches essais expérimentaux histologiques sur la gommose. Thèse au Laboratoire de Botanique et Biogéographie. Université Paul Sabatier de Toulouse, p 187

[CR22] Nussinovitch A (2009) Plant Gum Exudates of the World: Sources, Distribution, Properties, and Applications. Taylor and Francis. 10.1201/9781420052244

[CR23] Pomar F, Merino F, Barceló A (2002) *O*-4-linked coniferyl and sinapyl aldehydes in lignifying cell walls are the main targets of the Wiesner (phloroglucinol-HCl) reaction. Protoplasma 220:0017–0028. 10.1007/s00709-002-0030-y 10.1007/s00709-002-0030-y 12417933

[CR24] Prillieux E (1875) Etude sur la formation de la gomme dans les arbres fruitiers. Ann. des Sc. Nat. (bot.), 176p

[CR36] R Core Team (2017) R: A language and environment for statistical computing. https://www.Rproject.org/

[CR25] Saladino M, Kuhn H, Caianiello D, Robert F, Lusi, Basu A (2020) An Improved Protocol for the Synthesis and Purification of Yariv Reagents Raghuraj Hoshing. J. Org. Chem. 2020, 85, 16236 – 16242 10.1021/acs.joc.0c01812 33084327

[CR26] Schofield P, Mbugua DM, Pell AN (2001) Analysis of condensed tannins: a review. Anim Feed Sci Technol 91:1–2. 10.1016/S0377-8401(01)00228-0

[CR27] Société Française de Réalisation d’Études et de Conseils (SOFRECO) (2022) Analyse prospective de la chaine de valeur gomme arabique au Niger 2021–2030: Une dimension environnementale à valoriser

[CR28] Thevenet F (2009) Gomme d’acacia, hydrocolloïde multifonctionnel et nutritionnel. Additifs et adjuvants alimentaires. Techniques d’ingénieur. 27–28. 63p

[CR29] Torode TA, O’Neill R, Marcus SE, Cornuault V, Pose S, Lauder RP, Kračun SK, Rydahl MG, Andersen MC, Willats WG, Braybrook SA, Townsend BJ, Clausen MH, Knox JP (2018) Branched pectic galactan in phloem-sieve-element cell walls: implications for cell mechanics. Plant Physiol 176(2):1547–1558. 10.1104/pp.17.01568 29150558 10.1104/pp.17.01568 PMC5813576

[CR31] Vassal J, et Mouret M (1991) Etapes histologiques du processus de gommose chez *Acacia senegal*. In Physiologie des Arbres et Arbustes en zones arides et semi-arides. Groupe d’Étude de l’Arbre Ed, 277–281

[CR33] Yates EA, Valdor JF, Haslam SM, Morris HR, Dell A, Mackie W, Knox JP (1996) Characterization of carbohydrate structural features recognized by anti-arabinogalactan-protein monoclonal antibodies. Glycobiology 6(2):131–9. 10.1093/glycob/6.2.131 8727785 10.1093/glycob/6.2.131

[CR32] Yajun Liu L, Gao T, Xia, Zhao L (2009) Investigation of the Site-Specific Accumulation of Catechins in the Tea Plant (*Camellia sinensis* (L.) O. Kuntze) via Vanillin – HCl Staining. J Agricultural Food ChemistryVol 57/Issue 21. 10.1021/jf902614n 19831398

